# *N*,*N*′-Bis(3-methoxy­benzyl­idene)ethane-1,2-diamine

**DOI:** 10.1107/S1600536808026652

**Published:** 2008-08-23

**Authors:** Hoong-Kun Fun, Valiollah Mirkhani, Akbar Rostami Vartooni

**Affiliations:** a X-ray Crystallography Unit, School of Physics, Universiti Sains Malaysia, 11800 USM, Penang, Malaysia; b Chemistry Department, University of Isfahan, Isfahan 81746-73441, Iran

## Abstract

The mol­ecule of the title bidentate Schiff base ligand, C_18_H_20_N_2_O_2_, has twofold crystallographic rotation symmetry, giving one half-mol­ecule per asymmetric unit. It adopts a twisted *E* configuration with respect to the azomethine C=N bond. The imino group is coplanar with the aromatic ring. The dihedral angle between the two benzene rings is 69.52 (5)°. The meth­oxy group is coplanar with the benzene ring, as indicated by the C—O—C—C torsion angle of −179.56 (8)°. In the unit cell, mol­ecules are linked together by inter­molecular C—H⋯O hydrogen bonds, forming chains along the *a* axis; these chains are further stacked down the *b* axis by both inter­molecular C—H⋯O and C—H⋯π inter­actions.

## Related literature

For related structures see: Fun *et al.* (2008*a* ▶,*b* ▶,*c* ▶,*d* ▶); Calligaris & Randaccio, (1987 ▶). For information on Schiff base complexes and their applications, see: Kia *et al.* (2007*a* ▶,*b* ▶); Pal *et al.* (2005 ▶); Hou *et al.* (2001 ▶)
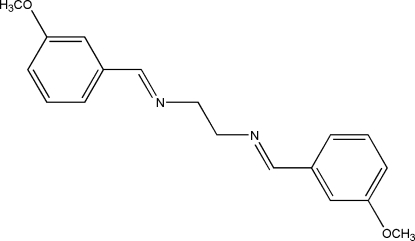


## Experimental

### 

#### Crystal data

C_18_H_20_N_2_O_2_
*M* *_r_* = 296.36
Monoclinic, 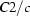

*a* = 22.7076 (3) Å
*b* = 6.0374 (1) Å
*c* = 11.6789 (2) Å
β = 100.235 (1)°
*V* = 1575.64 (4) Å^3^
*Z* = 4
Mo *K*α radiation
μ = 0.08 mm^−1^
*T* = 100.0 (1) K
0.49 × 0.33 × 0.22 mm

#### Data collection

Bruker SMART APEXII CCD area-detector diffractometer
Absorption correction: multi-scan (**SADABS**; Bruker, 2005 ▶) *T*_min_ = 0.886, *T*_max_ = 0.982
11683 measured reflections
2298 independent reflections
1879 reflections with *I* > 2σ(*I*)
*R*_int_ = 0.029

#### Refinement

*R*[*F* ^2^ > 2σ(*F* ^2^)] = 0.041
*wR*(*F* ^2^) = 0.108
*S* = 1.10
2298 reflections
113 parameters
H atoms treated by a mixture of independent and constrained refinement
Δρ_max_ = 0.35 e Å^−3^
Δρ_min_ = −0.21 e Å^−3^

### 

Data collection: *APEX2* (Bruker, 2005 ▶); cell refinement: *APEX2*; data reduction: *SAINT* (Bruker, 2005 ▶); program(s) used to solve structure: *SHELXTL* (Sheldrick, 2008 ▶); program(s) used to refine structure: *SHELXTL*; molecular graphics: *SHELXTL*; software used to prepare material for publication: *SHELXTL* and *PLATON* (Spek, 2003 ▶).

## Supplementary Material

Crystal structure: contains datablocks global, I. DOI: 10.1107/S1600536808026652/fl2216sup1.cif

Structure factors: contains datablocks I. DOI: 10.1107/S1600536808026652/fl2216Isup2.hkl

Additional supplementary materials:  crystallographic information; 3D view; checkCIF report

## Figures and Tables

**Table 1:** Hydrogen-bond geometry (Å, °)

*D*—H⋯*A*	*D*—H	H⋯*A*	*D*⋯*A*	*D*—H⋯*A*
C9—H9*A*⋯O1^i^	0.96	2.50	3.3809 (13)	153
C8—H8*B*⋯*Cg*1^ii^	0.984 (13)	2.822 (13)	3.6221 (12)	138.9 (9)
C9—H9*C*⋯*Cg*1^iii^	0.96	2.75	3.5636 (12)	143

Symmetry codes: (i) 
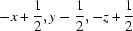
; (ii) 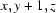; (iii) 
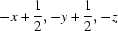
. *Cg*1 is the centroid of the C1–C6 benzene ring.

**Fig. 1.**
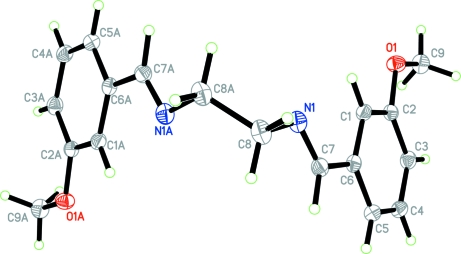
The molecular structure of (I) with atom labels and 50% probability ellipsoids for non-H atoms [symmetry code for A: -x + 1, Y, 0.5 - Z].

**Fig. 2.**
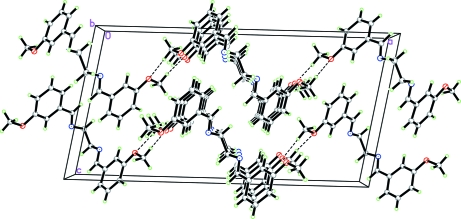
The crystal packing of (I), viewed down the b axis, showing chains along the a axis and stacking of these chains along the b axis. Intermolecular interactions are shown as dashed lines.

## supplementary crystallographic information

### Comment

Schiff bases are one of most prevalent mixed-donor ligands found in the field of coordination chemistry. There has been growing interest in Schiff base ligands, mainly because of their wide applications in the fields of biochemistry, synthesis, and catalysis (Kia *et al.*, 2007*a*,*b*; Pal *et al.*, 2005; Hou *et al.*, 2001). Many Schiff base complexes have been structurally characterized, but in comparison only a relatively small number of free Schiff bases have been described (Calligaris & Randaccio, 1987). As an extension of our work (Fun *et al.*, 2008*a*, 2008*b*, 2008*c*, 2008d) on the structural characterization of Schiff base compounds, the title compound (I), (Fig. 1), is reported here.

(I) has twofold crystallographic rotation symmetry to give 1/2 molecule per asymmetric unit  and it adopts a twisted *E* configuration with respect to the azomethine C=N bond. Bond lengths and angles are within normal ranges. The imino group is coplanar with the aromatic ring. The dihedral angle between two phenyl rings is 69.52 (5)°. The methoxy group is coplanar with the benzene ring as  indicated by the  C9–O1–C2–C1 torsion of  -179.56 (8)°. In the unit cell, (Fig. 2), neighbouring molecules are linked together by intermolecular C—H···O hydrogen bonds to form chains along the *a*-axis and these chains are further stacked down the *b*-axis by both intermolecular C—H···O and C—H···π interactions (Table 1).

### Experimental

The overall synthetic method has been described earlier (Fun *et al.*, 2008*a*), except that ethylenediamine (1 mmol, 60 mg) and 3-methoxybenzaldehyde (2 mmol, 137 mg) were used as starting materials. Single crystals suitable for *X*-ray diffraction were obtained by evaporation of an ethanol solution at room temperature.

### Refinement

H atoms bound to C7 and C8 were located from the difference Fourier map and freely refined. The rest of the hydrogen atoms were positioned geometrically with C—H = 0.93–0.96 Å and refined in riding mode with *U*_iso_(H) = 1.2 or 1.5 *U*_eq_(C). A rotating-group model was used for the methyl group.

### Crystal data

**Table:**

C_18_H_20_N_2_O_2_	*F*_000_ = 632
*M**_r_* = 296.36	*D*_x_ = 1.249 Mg m^−^^3^
Monoclinic, *C*2/*c*	Mo *K*α radiation λ = 0.71073 Å
Hall symbol: -C 2yc	Cell parameters from 3509 reflections
*a* = 22.7076 (3) Å	θ = 3.6–33.9º
*b* = 6.03740 (10) Å	µ = 0.08 mm^−^^1^
*c* = 11.6789 (2) Å	*T* = 100.0 (1) K
β = 100.2350 (10)º	Block, colourless
*V* = 1575.64 (4) Å^3^	0.49 × 0.33 × 0.22 mm
*Z* = 4	

### Data collection

**Table:**

Bruker SMART APEXII CCD area-detector diffractometer	2298 independent reflections
Radiation source: fine-focus sealed tube	1879 reflections with *I* > 2σ(*I*)
Monochromator: graphite	*R*_int_ = 0.029
*T* = 100.0(1) K	θ_max_ = 30.0º
φ and ω scans	θ_min_ = 3.5º
Absorption correction: multi-scan(*SADABS*; Bruker, 2005)	*h* = −31→31
*T*_min_ = 0.886, *T*_max_ = 0.982	*k* = −8→8
11683 measured reflections	*l* = −14→16

### Refinement

**Table:**

Refinement on *F*^2^	Secondary atom site location: difference Fourier map
Least-squares matrix: full	Hydrogen site location: inferred from neighbouring sites
*R*[*F*^2^ > 2σ(*F*^2^)] = 0.041	H atoms treated by a mixture of independent and constrained refinement
*wR*(*F*^2^) = 0.108	*w* = 1/[σ^2^(*F*_o_^2^) + (0.0467*P*)^2^ + 0.7792*P*] where *P* = (*F*_o_^2^ + 2*F*_c_^2^)/3
*S* = 1.11	(Δ/σ)_max_ < 0.001
2298 reflections	Δρ_max_ = 0.35 e Å^−^^3^
113 parameters	Δρ_min_ = −0.21 e Å^−^^3^
Primary atom site location: structure-invariant direct methods	Extinction correction: none

### Special details

**Table:**

Experimental. The low-temperature data was collected with the Oxford Cyrosystem Cobra low-temperature attachment.
Geometry. All e.s.d.'s (except the e.s.d. in the dihedral angle between two l.s. planes) are estimated using the full covariance matrix. The cell e.s.d.'s are taken into account individually in the estimation of e.s.d.'s in distances, angles and torsion angles; correlations between e.s.d.'s in cell parameters are only used when they are defined by crystal symmetry. An approximate (isotropic) treatment of cell e.s.d.'s is used for estimating e.s.d.'s involving l.s. planes.
Refinement. Refinement of *F*^2^ against ALL reflections. The weighted *R*-factor *wR* and goodness of fit *S* are based on *F*^2^, conventional *R*-factors *R* are based on *F*, with *F* set to zero for negative *F*^2^. The threshold expression of *F*^2^ > σ(*F*^2^) is used only for calculating *R*-factors(gt) *etc*. and is not relevant to the choice of reflections for refinement. *R*-factors based on *F*^2^ are statistically about twice as large as those based on *F*, and *R*- factors based on ALL data will be even larger.

### Fractional atomic coordinates and isotropic or equivalent isotropic displacement parameters (Å^2^)

**Table:**

	*x*	*y*	*z*	*U*_iso_*/*U*_eq_	
O1	0.29856 (3)	0.12810 (12)	0.16829 (6)	0.02029 (18)	
N1	0.44163 (4)	0.77578 (14)	0.17093 (8)	0.0197 (2)	
C1	0.36408 (4)	0.39310 (16)	0.11788 (8)	0.0166 (2)	
H1A	0.3629	0.4625	0.1885	0.020*	
C2	0.33032 (4)	0.20304 (16)	0.08715 (8)	0.0167 (2)	
C3	0.33099 (4)	0.10022 (17)	−0.01966 (9)	0.0196 (2)	
H3A	0.3080	−0.0257	−0.0407	0.024*	
C4	0.36637 (4)	0.18805 (18)	−0.09415 (9)	0.0216 (2)	
H4A	0.3671	0.1197	−0.1653	0.026*	
C5	0.40051 (4)	0.37574 (18)	−0.06388 (9)	0.0203 (2)	
H5A	0.4240	0.4328	−0.1145	0.024*	
C6	0.39970 (4)	0.47980 (17)	0.04289 (8)	0.0172 (2)	
C7	0.43794 (4)	0.67504 (17)	0.07480 (9)	0.0184 (2)	
C8	0.48159 (5)	0.96685 (17)	0.18898 (10)	0.0215 (2)	
C9	0.26352 (5)	−0.06749 (17)	0.14119 (10)	0.0225 (2)	
H9A	0.2443	−0.1047	0.2055	0.034*	
H9B	0.2890	−0.1874	0.1267	0.034*	
H9C	0.2337	−0.0419	0.0731	0.034*	
H7A	0.4614 (6)	0.722 (2)	0.0143 (11)	0.027 (3)*	
H8B	0.4562 (6)	1.100 (2)	0.1792 (11)	0.025 (3)*	
H8A	0.5085 (6)	0.971 (2)	0.1293 (12)	0.026 (3)*	

### Atomic displacement parameters (Å^2^)

**Table:**

	*U*^11^	*U*^22^	*U*^33^	*U*^12^	*U*^13^	*U*^23^
O1	0.0226 (4)	0.0197 (4)	0.0195 (4)	−0.0036 (3)	0.0063 (3)	−0.0004 (3)
N1	0.0174 (4)	0.0181 (4)	0.0228 (4)	−0.0004 (3)	0.0018 (3)	0.0020 (3)
C1	0.0170 (4)	0.0178 (4)	0.0144 (4)	0.0026 (3)	0.0015 (3)	−0.0002 (3)
C2	0.0152 (4)	0.0179 (4)	0.0167 (5)	0.0027 (3)	0.0020 (3)	0.0019 (3)
C3	0.0190 (5)	0.0191 (5)	0.0196 (5)	0.0007 (4)	0.0006 (4)	−0.0027 (4)
C4	0.0202 (5)	0.0281 (5)	0.0157 (5)	0.0031 (4)	0.0014 (4)	−0.0039 (4)
C5	0.0177 (5)	0.0271 (5)	0.0161 (5)	0.0012 (4)	0.0031 (4)	0.0016 (4)
C6	0.0153 (4)	0.0192 (5)	0.0163 (4)	0.0022 (3)	0.0003 (3)	0.0022 (4)
C7	0.0164 (4)	0.0197 (5)	0.0189 (5)	0.0008 (4)	0.0026 (4)	0.0056 (4)
C8	0.0186 (5)	0.0162 (5)	0.0291 (6)	−0.0010 (4)	0.0027 (4)	0.0027 (4)
C9	0.0226 (5)	0.0189 (5)	0.0255 (5)	−0.0033 (4)	0.0025 (4)	0.0019 (4)

### Geometric parameters (Å, °)

**Table:**

O1—C2	1.3659 (12)	C4—H4A	0.9300
O1—C9	1.4277 (12)	C5—C6	1.3993 (14)
N1—C7	1.2665 (14)	C5—H5A	0.9300
N1—C8	1.4597 (13)	C6—C7	1.4723 (14)
C1—C2	1.3912 (14)	C7—H7A	0.999 (13)
C1—C6	1.3954 (13)	C8—C8^i^	1.519 (2)
C1—H1A	0.9300	C8—H8B	0.984 (13)
C2—C3	1.3958 (14)	C8—H8A	1.005 (13)
C3—C4	1.3899 (14)	C9—H9A	0.9600
C3—H3A	0.9300	C9—H9B	0.9600
C4—C5	1.3830 (15)	C9—H9C	0.9600
			
C2—O1—C9	117.53 (8)	C1—C6—C7	121.54 (9)
C7—N1—C8	116.68 (9)	C5—C6—C7	118.98 (9)
C2—C1—C6	120.12 (9)	N1—C7—C6	123.50 (9)
C2—C1—H1A	119.9	N1—C7—H7A	122.1 (8)
C6—C1—H1A	119.9	C6—C7—H7A	114.4 (8)
O1—C2—C1	115.39 (8)	N1—C8—C8^i^	111.10 (7)
O1—C2—C3	124.31 (9)	N1—C8—H8B	106.9 (8)
C1—C2—C3	120.29 (9)	C8^i^—C8—H8B	108.8 (8)
C4—C3—C2	119.28 (9)	N1—C8—H8A	111.0 (8)
C4—C3—H3A	120.4	C8^i^—C8—H8A	110.5 (7)
C2—C3—H3A	120.4	H8B—C8—H8A	108.3 (11)
C5—C4—C3	120.84 (9)	O1—C9—H9A	109.5
C5—C4—H4A	119.6	O1—C9—H9B	109.5
C3—C4—H4A	119.6	H9A—C9—H9B	109.5
C4—C5—C6	120.01 (9)	O1—C9—H9C	109.5
C4—C5—H5A	120.0	H9A—C9—H9C	109.5
C6—C5—H5A	120.0	H9B—C9—H9C	109.5
C1—C6—C5	119.46 (9)		
			
C9—O1—C2—C1	−179.56 (8)	C2—C1—C6—C5	0.84 (14)
C9—O1—C2—C3	−0.45 (14)	C2—C1—C6—C7	−177.36 (8)
C6—C1—C2—O1	177.98 (8)	C4—C5—C6—C1	−0.24 (15)
C6—C1—C2—C3	−1.17 (14)	C4—C5—C6—C7	178.01 (9)
O1—C2—C3—C4	−178.19 (9)	C8—N1—C7—C6	−179.92 (9)
C1—C2—C3—C4	0.88 (15)	C1—C6—C7—N1	0.51 (15)
C2—C3—C4—C5	−0.27 (15)	C5—C6—C7—N1	−177.70 (10)
C3—C4—C5—C6	−0.04 (15)	C7—N1—C8—C8^i^	−136.92 (11)

Symmetry codes: (i) −*x*+1, *y*, −*z*+1/2.

### Hydrogen-bond geometry (Å, °)

**Table:**

*D*—H···*A*	*D*—H	H···*A*	*D*···*A*	*D*—H···*A*
C9—H9A···O1^ii^	0.96	2.50	3.3809 (13)	153
C8—H8B···Cg1^iii^	0.984 (13)	2.822 (13)	3.6221 (12)	138.9 (9)
C9—H9C···Cg1^iv^	0.96	2.75	3.5636 (12)	143

Symmetry codes: (ii) −*x*+1/2, *y*−1/2, −*z*+1/2; (iii) *x*, *y*+1, *z*; (iv) −*x*+1/2, −*y*+1/2, −*z*.
